# A Continuing Exploration of Tick–Virus Interactions Using Various Experimental Viral Infections of Hard Ticks

**DOI:** 10.3389/fphys.2018.01728

**Published:** 2018-12-04

**Authors:** Melbourne Rio Talactac, Emmanuel P. Hernandez, Kozo Fujisaki, Tetsuya Tanaka

**Affiliations:** ^1^Department of Clinical and Population Health, College of Veterinary Medicine and Biomedical Sciences, Cavite State University, Cavite, Philippines; ^2^Laboratory of Infectious Diseases, Joint Faculty of Veterinary Medicine, Kagoshima University, Kagoshima, Japan; ^3^National Agriculture and Food Research Organization, Tsukuba, Japan

**Keywords:** tick-borne viruses, ixodid ticks, virus infection, blood feeding, tick–virus interactions

## Abstract

To fully unravel the ixodid ticks’ role as vectors of viral pathogens, their susceptibility to new control measures, and their ability to develop acaricide resistance, acclimatization of ticks under laboratory conditions is greatly needed. However, the unique and complicated feeding behavior of these ticks compared to that of other hematophagous arthropods requires efficient and effective techniques to infect them with tick-borne viruses (TBVs). In addition, relatively expensive maintenance of animals for blood feeding and associated concerns about animal welfare critically limit our understanding of TBVs. This mini review aims to summarize the current knowledge about the artificial infection of hard ticks with viral pathogens, which is currently used to elucidate virus transmission and vector competence and to discover immune modulators related to tick–virus interactions. This review will also present the advantages and limitations of the current techniques for tick infection. Fortunately, new artificial techniques arise, and the limitations of current protocols are greatly reduced as researchers continuously improve, streamline, and standardize the laboratory procedures to lower cost and produce better adoptability. In summary, convenient and low-cost techniques to study the interactions between ticks and TBVs provide a great opportunity to identify new targets for the future control of TBVs.

## Introduction

Ticks are the most economically important vectors of livestock diseases (Arthur, 1962) and are considered second to mosquitoes in transmitting human diseases (de la Fuente et al., 2008; Socolovschi et al., 2009). Among the pathogens transmitted by these bloodsucking ectoparasites, tick-borne viruses (TBVs) present a severe health risk to both humans and domestic animals (Hoogstraal, 1973). TBVs comprise a wide range of viruses classified into eight virus families: *Asfarviridae*, *Nairoviridae, Peribunyaviridae, Phenuiviridae*, *Flaviviridae*, *Orthomyxoviridae*, *Rhabdoviridae*, and *Reoviridae* (Brackney and Armstrong, 2016; Kazimírová et al., 2017). Among these viral families, *Nairoviridae* and *Flaviviridae* are considered to have the TBVs of most importance to public health, including the tick-borne encephalitis virus (TBEV) and the Crimean–Congo hemorrhagic fever virus (CCHFV), which are known to cause severe clinical symptoms in humans (Nuttall et al., 1994; Labuda and Nuttall, 2004; Brackney and Armstrong, 2016; Kazimírová et al., 2017).

Of the 900 currently known tick species, less than 10% are implicated as virus vectors, and these include the *Ornithodoros* and *Argas* genera for the argasid ticks and *Ixodes*, *Haemaphysalis*, *Hyalomma*, *Amblyomma*, *Dermacentor*, and *Rhipicephalus* genera in ixodid ticks (Labuda and Nuttall, 2004; de la Fuente et al., 2017).

Although the role of ticks in the transmission of viruses has been known for over a century (Mansfield et al., 2017), the understanding of tick–virus interactions important for tick antiviral immunity, pathogen replication, and transmission of the virus to an animal host remains limited and at an early stage (Mitzel et al., 2007; Kopacek et al., 2010; Liu et al., 2012). Moreover, the diversity of tick-borne viruses has been less thoroughly studied than that of mosquito-borne viruses (Yoshii et al., 2015).

In addition, ixodid ticks differ essentially from other blood-feeding insects in terms of their digestive physiology, feeding behavior (Obenchain and Galun, 1982), and the long duration of the blood meal, which can take up to several weeks (Waladde and Rice, 1982). Moreover, tick attachment at feeding sites on the host requires correct physical and chemical stimuli for a successful engorgement (Guerin et al., 2000).

Since it is estimated that TBVs spend more than 95% of their life cycle within the tick vector (de la Fuente et al., 2017), a very intimate and highly specific association between tick vector species and the transmitted TBVs is normally maintained (Brites-Neto et al., 2015). With this in mind, artificial viral infection of ticks using experimental laboratory techniques can greatly improve our understanding of tick–virus interaction, particularly transmission pathways and vector competence. A comprehensive review of artificial tick infections using pathogens other than TBVs and the ixodid (hard) tick life cycle has already been made by Bonnet and Liu (2012). In this mini review, different techniques for the viral infection of hard ticks were presented, indicating their advantages and limitations with respect to their application to viral transmission and vector competency studies (summarized in Table 1).

**Table 1:** Summary of the techniques used to artificially infect ticks with representative ticks and viruses, their major advantages/disadvantages and associated references.

Tick-infection methods	Tick species	Virus studied	Main advantages	Main disadvantages
Direct feeding on infected host	*D. andersoni* *H. longicornis* *R. appendiculatus*	Powassan virus^1^ SFTS virus^2^ Thogoto virus^3^	Can infect a greater number of ticks; resembles the normal acquisition	Requires animal host; lacks quantification of acquired viral load
Co-feeding infection	*I. ricinus* *D. marginatus* *R. appendiculatus* *H. truncatum* *A. americanum* *H. longicornis*	TBEV^4-8^ Louping ill virus^9^ Bhanja virus^5^ Palma virus^5^ Thogoto virus^9,10^ CCHFV^11^ Heartland virus^12^ Thogoto virus^13^	An established natural viral infection of ticks	Requires animal host; greatly depends on the distance among feeding ticks
Membrane-feeding method	*I. ricinus* *I. ricinus* *D. reticulatus*	Bluetongue virus^14^ African swine fever virus^15^	Reduces variation within a given treatment group	Requires chemical and physical stimuli to enhance tick attachment; depends on the length of the hypostome; long attachment time
Capillary feeding	*A. variegatum* *R. appendiculatus* *I. ricinus* *D. reticulatus*	Dugbe virus^16^ Bluetongue virus^14^	Mimics the natural route of infection; can estimate the amount of introduced pathogen	Complicated maintenance of the integrity of the mouthparts of the ticks after removal
Percoxal injection	*H. longicornis* *A. variegatum*	Langat virus^17,18^ Thogoto virus^19^	Can estimate the amount of pathogen to be introduced	Requires a microinjector; may produce higher tick mortality due to injury
	*I. ricinus*	TBEV^4-6,8,20^		
		Louping ill virus^9^		
Anal pore injection	*H. truncatum* *I. ricinus*	CCHFV^21^ TBEV^19^		
	*H. longicornis*	Langat virus^17^		
Infection by immersion	*I. scapularis* *A. americanum*	LGTV^22,23^ Heartland virus^12^	Low cost; relatively simple artificial method; can synchronously infect ticks with a defined virus stock	May not generate cohorts of infected ticks with equal pathogen burden


*^1^Chernesky (1969), ^2^Luo et al. (2015), ^3^Booth et al. (1989), ^4^Labuda et al. (1996), ^5^Labuda et al. (1997a), ^6^Khasnatinov et al. (2009), ^7^Slovák et al. (2014), ^8^Labuda et al. (1993), ^9^Jones et al. (1997), ^10^Jones et al. (1987), ^11^Gordon et al. (1993), ^12^Godsey et al. (2016), ^13^Talactac et al. (2018), ^14^Bouwknegt et al. (2010), ^15^De carvalho Ferreira et al. (2014), ^16^Steele and Nuttall (1989), ^17^Talactac et al. (2016), ^18^Talactac et al. (2017b), ^19^Kaufman and Nuttall (1996), ^20^Belova et al. (2012), ^21^Gonzalez et al. (1989), ^22^McNally et al. (2012), and ^23^Tumban et al. (2011).*

## Methods for Infecting Ticks

### Direct Feeding on Infected Host

**FIGURE 1:**
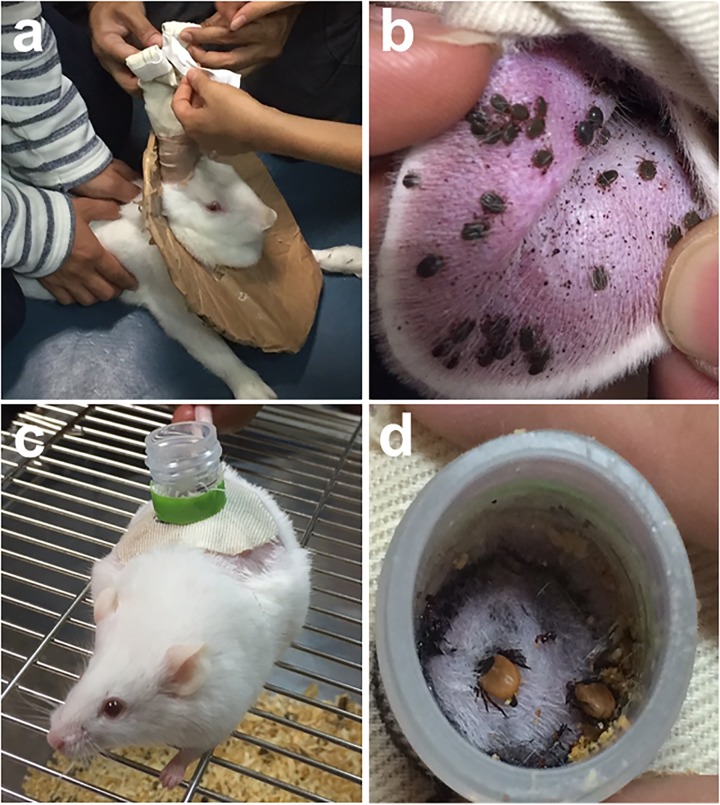
Feedings bags are glued or taped to clean-shaven ears of long-eared rabbit to allow tick engorgement **(a)**. *Haemaphysalis longicornis* nymphs infested on a rabbit’s ear **(b)**. A mouse with a feeding chamber attached on its back **(c)**. Ixodes persulcatus adult ticks infested on a mouse via chamber feeding **(d)**. Tick infestation using feeding bags or chamber feeders can be readily utilized to infect ticks with viruses via direct feeding on infected hosts.

Infesting ticks on infected natural hosts remains the method most closely resembling the normal acquisition of a virus in the wild. Direct feeding on infected host can be facilitated by using feeding bags (Figures 1a,b) or feeding chambers (Figures 1c,d). However, the maintenance and handling of animal hosts can be expensive and difficult, particularly for wild animals (Bonnet and Liu, 2012). The direct feeding technique also lacks the capacity to quantify the pathogen dose acquired by the tick during or post feeding. The technique may also not be appropriate for virus strains not suited for replication in the vertebrate hosts (Mitzel et al., 2007). In addition, it remains a challenge to synchronize viremia with tick feeding, and for ethical reasons, the use of alternative artificial methods in infecting ticks without the use of laboratory animals is still preferred (Bonnet and Liu, 2012).

Various hosts, mostly small laboratory animals, have already been infected for direct tick acquisition of the virus. *Dermacentor andersoni* ticks were previously infected by infesting rabbits injected intravenously with large doses of the Powassan virus (Chernesky, 1969). Laboratory mice were also previously used to study severe fever with thrombocytopenia syndrome virus (SFTSV) transmission by *Haemaphysalis longicornis* (Luo et al., 2015), while *Rhipicephalus appendiculatus* specimens were infected with the Thogoto virus (THOV) by allowing them to feed on THOV-infected Syrian hamsters (Booth et al., 1989). Transmission of West Nile virus from infected mice to naïve *I. ricinus* nymphs through direct blood feeding was also previously observed (Lawrie et al., 2004).

### Co-feeding Infection

**FIGURE 2:**
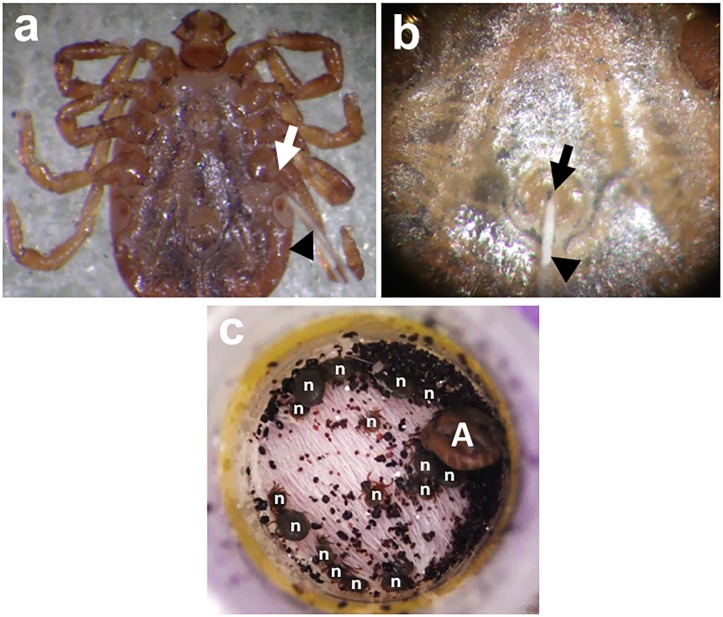
Percoxal **(a)** and anal pore/rectal **(b)** injections to *H. longicornis* adult ticks. Infection is accomplished by injecting the virus inoculum containing an estimated virus titer using glass microneedles (black arrowheads) at the joint of the tick coxa and trochanter of the 4th pair of legs (white arrow) or into the tick’s anal aperture (black arrow). After injection, ticks will be kept for 24 h in a 25°C incubator to observe for any mortality arising from possible injury due to the injection. Co-feeding **(c)** between a THOV-infected *H. longicornis* adult (A) and smaller uninfected/naïve nymphs (n). The ticks were infested on mice using feeding chamber/capsule method. All engorged nymphs post infestation will be collected and allowed to molt. Twenty-one days after molting, newly emerged adult ticks will be examined for either the presence of infectious virions or viral RNA using a focus formation assay or real-time PCR, respectively.

Non-viremic transmission, or co-feeding transmission (Figure 2c), is an important transmission mechanism for TBVs established by Jones et al. (1987). It occurs between infected and uninfected ticks when they co-feed in close proximity on susceptible hosts, even when these hosts do not develop viremia (Alekseev and Chunikhin, 1990; Labuda et al., 1993; Jones et al., 1997; Labuda et al., 1997a,b). Though co-feeding is an established natural tick infection method, it requires an animal host for feeding and may not produce high infection rates, as transmission of the virus from infected and uninfected ticks greatly depends on the proximity or distance among feeding ticks.

Co-feeding experiments were mostly conducted in small laboratory or wild animals. Virus transmission experiments using yellow-necked mice (*Apodemus flavicollis*) and bank voles (*Clethrionomys glareolus*) (Labuda et al., 1996, 1997b), BALB/c mice (Khasnatinov et al., 2009; Slovák et al., 2014), European hedgehog (*Erinaceus europaeus*), striped field mouse (*A. agrarius*) European pine vole (*Pitymys subterraneus*), and common pheasant (*Phaseanus colchicus*) (Labuda et al., 1993) were used to study TBEV transmission by *I. ricinus.*

Co-feeding transmission of the Louping ill virus on *I. ricinus* was also evaluated in mountain hares (*Lepus timidus*), New Zealand white rabbits (*Oryctolagus cuniculus*) and red deer (*Cervus elaphus*) (Jones et al., 1997). Non-viremic transmission was also established for Thogoto virus (THOV) on *R. appendiculatus* (Jones et al., 1987, 1997) and CCHFV on *Hyalomma truncatum*, *H. impeltatum* (Gordon et al., 1993) and *Amblyomma variegatum* (Gonzalez et al., 1991) using guinea pigs (*Cavia porcellus*). Co-feeding transmission was also observed for Bhanja virus and Palma virus in *D. marginatus, D. reticulatus*, and *I. ricinus* ticks infested on mice (Labuda et al., 1997a) and Heartland virus in *Amblyomma americanum* infested on rabbits (Godsey et al., 2016). Lastly, co-feeding transmission of THOV was recently demonstrated in *H. longicornis* ticks infested on BALB/c mice (Talactac et al., 2018).

### Membrane-Feeding Methods

Another alternative to tick infestation is through membrane feeding. Membranes from animal and non-animal origin (e.g., silicone membranes) are usually utilized, with variable success, to feed ticks. This method could also be used for studies on the dynamics of pathogen transmission, since it can reduce the variation within a given treatment group because the blood meal from the same donor reduces the variation that may arise from individual tick–host relationships (Krober and Guerin, 2007).

However, this method requires chemical and physical stimuli to enhance attachment by hard ticks to membranes (Kuhnert, 1996). Its use may also depend on the length of the hypostome in all life stages of the hard ticks to be studied (Krober and Guerin, 2007). In addition, this type of artificial feeding is more challenging for ixodid ticks, since they require longer time for attachment (de Moura et al., 1997. This method was previously used in infecting *I. ricinus*, *I. hexagonus*, *D. reticulatus*, and *R. bursa* with the Bluetongue virus (Bouwknegt et al., 2010). The unlikely involvement of *I. ricinus* and *D. reticulatus* as biological vectors of African swine fever virus was also shown using membrane feeding (De carvalho Ferreira et al., 2014).

### Infection Through Capillary Feeding

The introduction of pathogens to ixodid ticks via capillary feeding was first attempted by Chabaud (1950). In this technique, the ticks are normally pre-fed on animals, followed by a careful mechanical removal of ticks from the host. Eventually, a capillary tube containing the pathogen is placed over the tick’s mouthparts, and the tick is immobilized on a slide (Burgdorfer, 1957; Bouwknegt et al., 2010). Capillary feeding provides a number of advantages, especially that it mimics the natural route of infection of ticks, and it can estimate the amount of pathogen to be introduced. However, maintaining the integrity of the mouthparts of the ticks after removal is crucial for a successful capillary feeding (Bonnet and Liu, 2012). This technique was previously used in infecting *A. variegatum* and *R. appendiculatus* with the Dugbe virus (Steele and Nuttall, 1989) and *I. ricinus*, *I. hexagonus*, *D. reticulatus*, and *R. bursa* with the Bluetongue virus (Bouwknegt et al., 2010).

### Infection Through Injection

Direct injection of the virus inoculum through the cuticle (between the coxa and trochanter) has the advantage of estimating the viral dose received by the ticks (Figure 2a). However, this method bypasses the midgut barrier of ticks during feeding, making it unrepresentative of the natural route of infection for ticks (Mitzel et al., 2007). This technique also requires a microinjector to efficiently introduce the inoculum into the tick and may produce higher tick mortality due to injection injury (Rechav et al., 1999). Previous studies using this technique include the infection of *H. longicornis* with the Langat virus (Talactac et al., 2016, 2017a), *A. variegatum* with the Thogoto virus (Kaufman and Nuttall, 1996), *I. ricinus* with TBEV (Labuda et al., 1993, 1996, 1997b; Khasnatinov et al., 2009; Belova et al., 2012), and the Louping ill virus (Jones et al., 1997). *D. marginatus*, *D. reticulatus*, and *I. ricinus* ticks also previously received percoxal injections with the Bhanja and Palma viruses (Labuda et al., 1997a). Alternatively, anal pore or rectal injection of the virus directly into the gut of the tick can be used (Figure 2b), though it also requires skill to avoid puncturing the gut upon injection. This method has been used to infect *H. truncatum* with CCHFV (Gonzalez et al., 1989), *I. ricinus* with TBEV (Belova et al., 2012), and *H. longicornis* with Langat virus (Talactac et al., 2017b) and THOV (Talactac et al., 2018).

### Infection Through Immersion

Infection of ticks through immersion provides a low cost and relatively simple artificial method, since it can synchronously infect a large number of ticks with a defined virus stock. The ticks are believed to be infected when they successfully swallowed the immersion medium containing the virus; with the ingested virus ultimately reaching the midgut (Mitzel et al., 2007). The virus can also possibly penetrate the tick’s exoskeletons, especially the immature ones. Larvae and nymphs have less sturdy exoskeleton, since arthropods must be able to hydrolyze the chitin for cuticle degradation and development during the immature stages (You et al., 2003). However, its major limitation is the generation of cohorts of infected ticks with an equal pathogen burden (Kariu et al., 2011). Infection of ticks using this method was previously reported for *I. scapularis* infected with Langat virus (Tumban et al., 2011; McNally et al., 2012), the dengue virus (Tumban et al., 2011), and TBEV (Mitzel et al., 2007) and for *A. americanum* infected with the Heartland virus (Godsey et al., 2016).

## Conclusion

To fully understand the interaction of ticks with TBVs, efficient techniques for the artificial infection and maintenance of tick colonies under laboratory conditions are crucial. As emphasized in this mini review, it is the unique but complicated feeding behavior of ixodid ticks that makes studies related to virus transmission, vector competence, and other aspects of tick–virus interaction a challenging endeavor. However, with the availability of these alternative feeding methods and techniques to infect ticks with different viruses of public health importance, the potential for studies on TBVs to catch up with the advances in mosquito-borne viral disease research is no longer a far-fetched scenario. In addition, the limitations of current techniques do not outweigh importance of studying TBVs. Understanding the interactions between ticks and the TBVs they transmit offers a great opportunity to identify new targets for the future control of TBVs.

## Author Contributions

All authors listed have made a substantial, direct and intellectual contribution to the work, and approved it for publication.

## Conflict of Interest Statement

The authors declare that the research was conducted in the absence of any commercial or financial relationships that could be construed as a potential conflict of interest.
